# Research on a Hybrid Scheduling Algorithm Based on Critical-Link Optimization for Large-Scale Time-Triggered Ethernet

**DOI:** 10.3390/s25206347

**Published:** 2025-10-14

**Authors:** Haowen Zhu, Zhen Li, Jinwei Cheng, Zhonghe Jin

**Affiliations:** 1 School of Aeronautics and Astronautics, Zhejiang University, Hangzhou 310058, China; haowenzhu2006@126.com; 2 Shanghai Aerospace Electronic Technology Institute, Shanghai 201108, China; lizhen202501@126.com (Z.L.); 18952523630@163.com (J.C.)

**Keywords:** scheduling algorithm, critical-link optimization, Time-Triggered Ethernet

## Abstract

With the rapid development of the Industrial Internet of Things (IIoT), the application scale of Time-Triggered Ethernet (TTE) technology in the IIoT has been increasingly expanding. To address the issues of rapidly increasing computation time and deteriorating scheduling quality in traditional scheduling algorithms for large-scale TTE applications, this paper proposes a hybrid scheduling algorithm based on critical-link optimization. A large-scale TTE message scheduling model is established based on the characteristics of Time-Triggered (TT) messages, and the constraints of TT scheduling are mathematically abstracted. After identifying the critical link of the network, a time slot balancing scheduling algorithm based on static priority is adopted for the link. The algorithm searches for the optimal scheduling time of current message by time-sliding within the current maximum time gap of TT messages from the center to both sides, maximizing the balance of TT message intervals to reduce the impact on Best-Effort (BE) message transmission performance. An improved genetic algorithm is proposed for the scheduling of the entire network to further enhance the global optimization capability, which takes the scheduling results of the critical link as the genes of initial population. The TT scheduling constraints are converted into the fitness function and the optimized genetic operators are developed for the genetic algorithm. Simulation results showed that the proposed algorithm can significantly reduce computing time and increase the success rate of message scheduling. At the same time, the scheduling results exhibit a better degree of TT message balance and can effectively reduce the transmission delay and jitter of BE messages as message load increases compared with traditional algorithms, making it better meet the scheduling requirements of large-scale TTE application scenarios.

## 1. Introduction

Building on next-generation Industrial Internet of Things (IIoT) technology, the Industrial 5.0 era aims to establish a fully interconnected industrial network system that connects humans, machines, and things [1]. This system enables real-time acquisition, free transmission, precise analysis, and intelligent feedback of massive industrial data. With the development of IIoT, an increasing number of devices are interconnected across networks, requiring diverse data traffic to be supported in a single network simultaneously, such as high-security control, high-bandwidth AI perception, and real-time cross-domain human–machine interaction. The traditional “best-effort” mechanism of Ethernet is no longer sufficient to meet the requirements of microsecond-level synchronization and mixed deterministic transmission [2].

Time-Triggered Ethernet (TTE) extends conventional Ethernet by incorporating time-triggering, synchronization, and redundancy mechanisms [3,4]. TTE can serve applications with varying latency and reliability requirements over the same physical network, providing sub-microsecond-level time synchronization and bounded transmission jitter using a global clock and offline schedule tables. TTE also supports fault tolerance and traffic isolation while remaining fully compatible with traditional Ethernet. This makes it well-suited for the stringent transmission and processing demands of industrial networks. TTE technology has gained widespread attention and recognition in the industry and is increasingly being applied in fields such as healthcare, aerospace, and automotive [5,6,7,8].

TTE classifies traffic into Time-Triggered (TT) and Event-Triggered (ET) traffic [9], with the latter comprising Rate-Constrained (RC) and Best-Effort (BE) messages. TT traffic is transmitted according to a pre-defined schedule, ensuring deterministic latency and jitter, while ET traffic is transmitted in the gaps between TT transmissions. Consequently, the quality of the TT scheduling table directly impacts both TT traffic performance and the transmission of other traffic types, thereby influencing the overall network capacity [10]. With the large-scale deployment of TTE, network topologies have become increasingly complex. The demand for real-time data transmission has grown substantially, resulting in greater computational complexity in solving TTE scheduling problems [11]. Therefore, in large-scale TTE networks, how to rapidly deliver high-quality solutions tailored to specific applications and operational requirements remains a pressing challenge for TTE scheduling algorithms.

The solving of TTE scheduling tables is NP-Complete Problem (NPC). In current research and engineering applications, traditional TTE scheduling methods mainly include Satisfiability Modulo Theory (SMT) -based algorithms [12] and Metaheuristic Algorithms (MA) [13]. In [14], a network-planning-based SMT solver was first proposed to generate static schedules, and its performance was validated through simulation. An incremental scheduling mechanism was also introduced, in which TT traffic is divided into multiple batches and schedules are generated sequentially using the SMT solver; however, the results yielded only feasible solutions, with limited improvement in bandwidth and time utilization. In [15], the authors introduced the Strict Periodic Utilization (SPU) factor to quantify the scheduling difficulty of TT traffic, and TT flows are then incrementally scheduled by the SMT solver in descending order of SPU. By computing interference times between TT flows, the scale of conflict-free constraints between scheduled and unscheduled flows during incremental scheduling is reduced, thereby decreasing the number of backtracking steps. In [16], a load-balancing strategy and distributed iterative conflict backtracking method were incorporated into the SMT framework, where message clusters were divided into smaller subsets and each subset was solved once at a time. When a subset was successfully scheduled, the results were added as new constraints to the SMT solver, improving both computational efficiency and load balance to some extent. In [17], a Fuzzy-controlled Quantum-behaved Particle Swarm Optimization (FQPSO)-SMT algorithm was proposed, in which priorities were defined to determine the order of incremental scheduling. This method considered not only end-to-end delay but also interference between scheduled and unscheduled messages, enabling rapid resolution of collision-free and timing constraint problems for basic periodic frames, with better load balancing and bandwidth utilization than standard incremental SMT. In [18], a hybrid scheduling technique combining Genetic Algorithm (GA) and Simulated Annealing (SA) was employed, where chromosome encoding, a penalty-based fitness function, and elite selection with POX crossover were designed, and by incorporating a simulated-annealing strategy during the mutation phase, the algorithm enhances both global and local search capabilities. However, this approach was limited to smaller network topologies and message sets, with SA parameters empirically tuned and lacking adaptive mechanisms.

Since TT messages have the highest scheduling priority in the network, the results of TTE scheduling algorithms have a significant impact on the performance of ET messages. In [19], the paper introduced a novel Dynamic Programming Priority (DPP) algorithm designed for scheduling RC flows in TTE, which combines priority-based scheduling with dynamic programming, dividing RC flows into different priority groups. Higher-priority groups are scheduled using First input first output (FIFO) policy, while lower-priority groups leverage dynamic programming for optimal resource utilization. The algorithm also integrates an SMT solver to pre-schedule TT messages, minimizing their interference with RC flows. In [20], the authors first employed an SMT solver to obtain an initial schedule where TT traffic is distributed as evenly as possible while satisfying inherent transmission constraints. Network calculus was then applied to verify whether RC traffic met its delay requirements; if satisfied, the scheduling process terminated with the current result, otherwise, TT flows causing excessive RC traffic delays were rescheduled until the RC traffic delay requirement was met. In [21], a Modified Weighted Round-Robin (MWRR) scheduling algorithm based on optimal time slices was proposed. The algorithm utilizes SMT to generate an offline scheduling table for TT messages, thereby optimizing the transmission time slices for RC messages. Within these time slices, bandwidth is allocated proportionally and a deficit counter is introduced to ensure fairness, thus improving the scheduling fairness and real-time performance of different types of RC messages. In [22], A TTE optimal scheduling technology based on rapid increment was proposed to optimize scheduling intervals to reduce RC message waiting time. By backtracking to correct multi-hop delays and adjusting the TT message schedule, the transmission performance of RC messages is improved. In [23], a scheduling optimization method was introduced to minimize the “makespan” of TT messages, aiming to reserve the maximum possible bandwidth for RC traffic to ensure its real-time performance and stability. However, this model imposes strict constraints on link bandwidth and message periods, and its computational complexity increases sharply with the number of messages, limiting its practical applicability.

Based on the current research on TTE scheduling algorithms, several pressing issues remain when addressing large-scale TTE applications. First, due to factors such as sensor locations, service characteristics, network topology, and resource distribution, traffic loads vary significantly across different links within the same network [24,25]. Traditional scheduling algorithms typically employ unified global scheduling strategies without accounting for the traffic heterogeneity of different links, which reduces computational efficiency and limits the quality of the solutions. Second, in practical applications, BE messages often constitute the majority of traffic in TTE networks. Real-time and precise remote monitoring and control rely on stable BE traffic (such as image and audio) to support human–machine feedback interaction. Since BE messages have the lowest priority, the scheduling results for TT messages have the greatest impact on the performance of transmission of BE messages; however, existing optimization efforts primarily focus on the scheduling of TT and RC messages, while the impact on BE messages has not been a primary research focus. Finally, the complexity of the network topology and the number of network messages that need to be scheduled increase rapidly as the scale of the TTE network expands. The issue of the time complexity of traditional algorithms becomes increasingly critical, making it difficult to obtain feasible solutions even in certain complex scenarios [26].

To address the above issues, this paper proposes a hybrid scheduling algorithm based on critical-link optimization for large-scale TTE. First, a TT network topology and message scheduling model is established based on large-scale network application scenarios, and the constraints of TT scheduling are analyzed and mathematically abstracted. Second, a slot-balanced scheduling algorithm based on static priority is introduced for the most critical link, in which messages on the link are first prioritized according to their periods, and then scheduled sequentially by priority. For each message, the scheduling slot is determined by searching the feasible optimal slot closest to the center of the largest TT message gap according to the distribution of already scheduled messages to achieve the most balanced time slot arrangement, thus reducing the impact of continuous TT slot allocation on delay and jitter of BE message transmission. Finally, after completing the critical link scheduling, a genetic algorithm is applied to solve the network-wide scheduling problem and the scheduling results of the critical link are used as input to the genetic algorithm. The scheduling constraints are converted into the fitness function of the genetic algorithm and optimized genetic operators are used to further improve the algorithm’s optimization capabilities. By combining static scheduling for the critical link with dynamic global scheduling based on genetic algorithm, the proposed hybrid scheduling algorithm effectively reduces computation time while improving scheduling quality.

## 2. Time-Triggered Ethernet Model

### 2.1. Network Traffic Analysis

TTE supports diverse types of data transmission, with its services built upon network-wide time synchronization. It enables the coexistence of three distinct classes of message transmission over a single physical network [27]. TT messages are subject to strict temporal constraints. Their transmission times are determined by a pre-defined offline communication schedule and are strictly executed at scheduled time points during runtime. This schedule is cyclically repeated with a fixed duration known as the cluster cycle [28]. RC messages are ET messages regulated through bandwidth allocation mechanisms. These messages achieve flow control and data rate limitation via the Bandwidth Allocation Gap (BAG). BE messages are also ET messages but are not subject to any timing or bandwidth constraints. Traditional Ethernet traffic falls under the BE message category.

**Figure 1:**
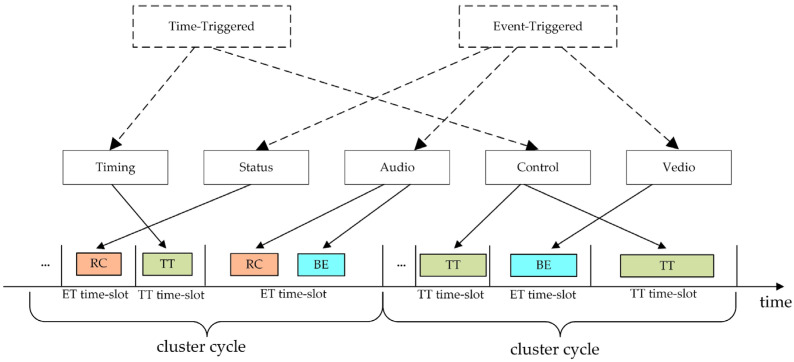
Mixed scheduling in a TTE network.

In TTE, different applications typically employ different message types according to their transmission requirements. The network performs mixed scheduling and transmission of the three message types based on an offline scheduling table. For example, in aerospace vehicle networks, critical data such as real-time control and time services are transmitted using TT messages, while status data, voice, video, and other services are transmitted using RC and BE messages. An illustration of mixed scheduling in a TTE network is shown in Figure 1.

In TTE, since BE traffic can only be transmitted during the idle intervals of TT and RC traffic, the scheduling of TT messages increasingly impacts the performance of BE traffic as the network scales up. The significant delay and jitter experienced by BE messages can adversely affect applications relying on BE traffic, such as causing stuttering or latency in video or voice communications, as well as data congestion and packet loss. These issues, in turn, constrain the overall performance of the TTE network. Therefore, optimizing the static scheduling table of TT messages to reduce their interference with BE traffic is crucial for improving the overall performance of TTE networks.

### 2.2. Network Topology Model

**Figure 2:**
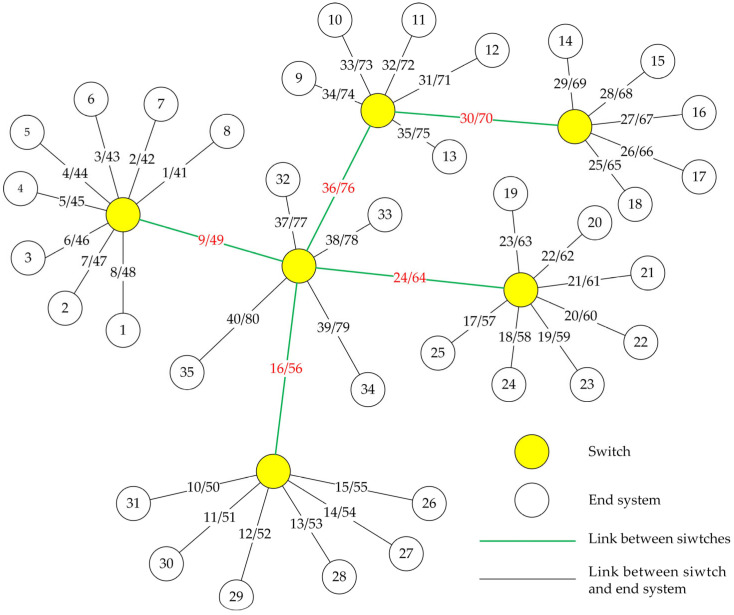
Topology of large-scale multi-hop TTE network.

Traditional TTE networks typically employ simple topologies such as star or snowflake structures. To evaluate the performance of algorithms under large-scale and non-single-path network conditions, this study constructs a multi-hop tree topology based on a TTE network comprising 35 end systems and 6 switches. The network topology is illustrated in Figure 2.

In the figure, circles represent network end systems or switch nodes, while the lines between nodes represent network links. The multi-hop TTE network is modeled as an undirected graph G(V,E), where the vertex set V (i.e., the circles in the figure) represents switches or end systems, and the edge set E (i.e., the straight lines) denotes bidirectional connections between vertices. Let F denote the set of all frames in the network. A vector L represents the set of data links, where $(v_{x},v_{y})\in L$ indicates a data link directed from vertex $v_{x}$ to vertex $v_{y}$, and $(v_{y},v_{x})\in L$ denotes the data link in the opposite direction on the same edge. Two vertices $(v_{x},v_{y})\in V$ can exchange frames only if at least one of them is a switch.

### 2.3. Message Scheduling Model

**Figure 3:**
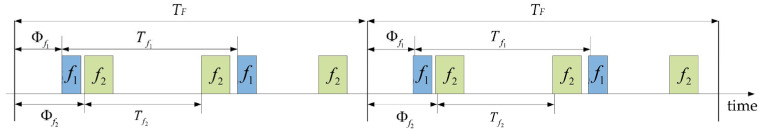
Periodic scheduling model of TT messages.

In a TTE network, all TT frames are transmitted periodically, though the transmission periods may differ among virtual link messages. Suppose there are N messages in the network, and $T_{f_{n}}$ denotes the transmission period of frame $f_{n}$. The cluster cycle, denoted as $T_{F}$, is defined as the least common multiple (LCM) of the periods $T_{f_{1}}$, $T_{f_{2}}$, …,$T_{f_{n}}$ of all TT frames. The variable $\Phi_{f_{n}}$ represents the offset of frame $f_{n}$ relative to the start of the cluster cycle. A schematic diagram of the TT message periodic scheduling model is shown in Figure 3, which illustrates two TT messages.

Figure 3 illustrates the periodic transmission characteristics of two TT messages, $f_{1}$ and $f_{2}$, over two cluster cycles. As shown, within each cluster cycle, the same TT message maintains a consistent time offset relative to the start of the cycle. Since TT traffic is deterministic and periodic, once the offset of a message is determined, its transmission schedule over time can be derived based on its period. Therefore, the TT message scheduling problem can be simplified to determining the time offsets of all messages within a single cluster cycle. This leads to the construction of a static communication schedule table that ensures collision-free transmission of TT messages over the network links [29]. After the schedule table is generated, the configuration tool encapsulates the schedule table into an image file and loads it into the local storage of each switch and end device before the network starts to run. After the schedule table is loaded, each switch and terminal transmits and receives data according to the locally stored scheduling table.

The time offset of a frame within a single cluster cycle is defined as follows:

(1) $$\Phi_{f}(i,l,j)=t,t>0$$

It indicates that the source node j begins transmitting the i-th frame over link l at time t after the start of the cluster cycle. According to the periodic nature of TT messages, the interval between the i-th frame and the i+1-th frame is equal to the frame’s period. This relationship can be expressed mathematically as follows:

(2) $$\Phi_{f}(i+1,l,j)=\Phi_{f}(i,l,j)+T_{f},i=1,2,\cdots N_{f}$$

An end system transmitter can send information to one or multiple receiving end systems. Therefore, in a TT network architecture, unicast, broadcast, and multicast communications can be implemented at the link layer. The data flow path $p_{i}$ is defined as the sequence of links from the sender $v_{s}$ to the receiver $v_{r}$, as shown in following:

(3) $$p_{i}=[(v_{s},v_{s+1}),\cdots ,(v_{r-1},v_{r})]$$

For any frame f, its path tree is defined as $TP_{f}$, which is the union of all data flows from the sender of frame f to each of its receivers. Accordingly, frame f can be defined by a tuple as shown in following:

(4) $$f=\langle T_{f},\Phi_{f},L_{f},{TP}_{f}\rangle$$

In this tuple, $T_{f}$ denotes the transmission period of the frame, $\Phi_{f}$ represents the time offset of the frame, $L_{f}$ is the frame length (in bytes), and $TP_{f}$ is the path tree as defined above. Based on the previous definitions, for a given set of frames F, the message scheduling table can be constructed using the set of time offsets $\Phi_{f}$ to form a complete transmission schedule.

Since searching for time offsets in the continuous time domain is particularly complex, the continuous transmission time is discretized into natural-number-based time slots. A basic time slot unit is defined as 1 microsecond and is denoted by $t_{size}$. Thus, both the transmission offset and duration of each frame can be expressed in terms of time slots, which simplifies the problem. Under this assumption, the transmission duration of frame f on link l can be represented by the number of time slots $\delta_{f}^{l}$, as shown in following:

(5) $$\delta_{f}^{l}=\frac{t_{f}^{l}}{t_{size}}$$

Here, $t_{f}^{l}$ represents the actual transmission duration of frame f on link l, and other time-related variables can be similarly expressed and simplified using this time slot representation.

Considering that the cluster cycle $T_{F}$ is the LCM of a set of frame periods, a frame on a given virtual link may need to be scheduled multiple times within a single cluster cycle. Therefore, the number of scheduling instances is calculated as follows:

(6) $$N_{f}=\frac{T_{F}}{T_{f}}$$

In the formula, $T_{f}$ represents the period of frame f.

## 3. Time-Triggered Scheduling Constraints

### 3.1. Frame Period Constraint

For any frame f, in order to ensure that the time offsets of all links along the path tree satisfy the frame period constraint, the following condition must be met: the deadline of the first instance of frame f on any link l within the path tree shall fall within the time interval $\left(0,T_{f}\right]$. The frame period constraint is mathematically expressed as follows:

(7) $$\begin{array}{l} \forall f\in F,\;\;\;\forall l\in TP_{f}:\\ 0<\Phi_{f}(1,l,j)+\delta_{f}^{l}\leq T_{f} \end{array}$$

In the formula, F represents the set of all frames in the network, $TP_{f}$ is the path tree of frame f, and $\delta_{f}^{l}$ is the transmission duration of frame f on link l. Once the period constraint is satisfied, the time offset of the i-th frame transmitted from the source node can be derived based on the offset of the first frame. The calculation is given as follows:

(8) $$\Phi_{f}(i,l,j)=\Phi_{f}(1,l,j)+(i-1)\times T_{f}$$

### 3.2. Contention-Free Constraint

The contention-free constraint ensures that no data link conflict occurs between any two TT message frames, and it is the most fundamental requirement in a TTE static schedule table. In other words, the transmission time of any frame on any link within the network system cannot overlap. The contention-free constraint is formally described by the mathematical expression as follows:

(9) $$\begin{array}{l} \forall (v_{x},v_{y})\in L,\forall f_{m},f_{n}\in F:\\ (f_{m}\neq f_{n}),i_{f_{m}}=1,2\cdots N_{f_{m}},i_{f_{n}}=1,2\cdots N_{f_{n}}\Rightarrow \\ (\Phi_{f_{m}}(i_{f_{m}},(v_{x},v_{y}),v_{x})\geq \Phi_{f_{n}}(i_{f_{n}},(v_{x},v_{y}),v_{x})+\delta_{f_{n}}^{l})\vee \\ \left(\Phi_{f_{n}}(i_{f_{n}},(v_{x},v_{y}),v_{x})\geq \Phi_{f_{m}}(i_{f_{m}},(v_{x},v_{y}),v_{x})+\delta_{f_{m}}^{l}\right) \end{array}$$

In the formula, F represents the set of all frames in the network, $f_{m}$ and $f_{n}$ represent any two distinct frames. $\delta_{f_{m}}^{l}$ and $\delta_{f_{n}}^{l}$ represent the transmission duration of frames on link l. Since TT message frames exhibit periodic behavior, Equation (9) can be further simplified to Equation (10), where $T_{F}$ denotes the LCM of the periods of all frames, as described earlier.

(10) $$\begin{array}{l} \forall (v_{x},v_{y})\in L,\forall f_{m},f_{n}\in F(f_{m}\neq f_{n}),\\ \forall a\in \left[0,1\cdots (\frac{T_{F}}{T_{f_{m}}}-1)\right],\forall b\in \left[0,1\cdots (\frac{T_{F}}{T_{f_{n}}}-1)\right]:\\ \left(a\times T_{f_{m}}+\Phi_{f_{m}}(1,(v_{x},v_{y}),v_{x})\geq b\times T_{f_{n}}+\Phi_{f}(1,(v_{x},v_{y}),v_{x})+\delta_{f_{n}}^{l}\right)\vee \\ \left(b\times T_{f_{n}}+\Phi_{f_{n}}(1,(v_{x},v_{y}),v_{x})\geq a\times T_{f_{m}}+\Phi_{f_{m}}(1,(v_{x},v_{y}),v_{x})+\delta_{f_{m}}^{l}\right) \end{array}$$

Here, a and b denote the sequence numbers of frames $f_{m}$ and $f_{n}$ on the link, respectively—that is, the a-th instance of $f_{m}$ and the b-th instance of $f_{n}$ on the link.

### 3.3. Causality Constraint

The data flow path of a frame must follow the transmission order of links from the sender to the receiver. Therefore, a switch must first receive a frame before it can forward it. The causality constraint ensures the correct temporal ordering of TT frame transmissions across adjacent physical links involving a switching node. Specifically, for each pair of consecutive links $(v_{x},v_{y}),(v_{y},v_{x})\in TP_{f}$, which belong to the path tree of frame f, the causality constraint as the frame passes through the relay node can be described as follows:

(11) $$\begin{array}{l} \forall p_{i}\in TP_{f},\forall (v_{x},v_{y}),(v_{y},v_{z})\in p_{i}:\\ \left(\Phi_{f}(i,(v_{y},v_{z}),v_{y})-\Phi_{f}(i,(v_{x},v_{y}),v_{x})\right)\geq T_{hd} \end{array}$$

Here, $T_{hd}$ represents the minimum time a frame is stored in the switch before being relayed—that is, the minimum forwarding delay of the switch.

### 3.4. Buffer Capacity Limit Constraint

The scheduling algorithm must also consider the Buffer capacity limit of relay switches—that is, the maximum duration a TT frame can be buffered within a switch. If this upper bound is exceeded, packet loss may occur. Therefore, for each pair of consecutive links $(v_{x},v_{y}),(v_{y},v_{z})\in TP_{f}$, which belong to the path tree of frame f, the buffer capacity limit constraint as the frame passes through the relay node can be expressed as follows:

(12) $$\begin{array}{l} \forall p_{i}\in TP_{f},\forall (v_{x},v_{y}),(v_{y},v_{z})\in p_{i}:\\ \left(\Phi_{f}(i,(v_{y},v_{z}),j)-\Phi_{f}(i,(v_{x},v_{y}),j)\right)\leq T_{mm} \end{array}$$

$T_{mm}$ denotes a constant determined by switch memory size, representing the maximum buffering time before frame forwarding. Unlike non-TT traffic, output port contention is not considered, allowing queuing analysis to be omitted.

### 3.5. End-to-End Delay Constraint

For TT message transmission, the time interval from the start of frame transmission at the sender to its reception at each receiver must be bounded. Although such end-to-end delay is practically constrained by buffer overflow avoidance, it can be further restricted by the parameter $T_{et}$. The end-to-end delay constraint for a data frame f, from the transmitting node to the receiving node, is defined as follows:

(13) $$\left(\Phi_{f}(i,(v_{r-1},v_{r}),j)-\Phi_{f}(i,(v_{s},v_{s+1}),j)\right)<T_{et}$$

In this equation, $v_{r}$ denotes the destination node of the message in the network topology, and $v_{s}$ represents the source node.

## 4. Scheduling Algorithm Based on Critical-Link Optimization

### 4.1. Algorithm Procedure and Analysis

#### 4.1.1. Algorithm Procedure

The message planning and scheduling of the TTE network usually follows a two-step process. The first step is message path planning, which determines the transmission links for each message. The second step is message time-slot scheduling, which allocates transmission time slots for each message on the corresponding link. The algorithm proposed in this paper focuses on the second step and is carried out based on the following premises: the network topology structure is predetermined and does not involve dynamic topology changes; the transmission paths of all TT messages have been determined through offline planning or preset mechanisms, without considering dynamic routing adjustments, path reselection, and path distance constraints. On this basis, this study focuses on efficient slot allocation and conflict avoidance for TT messages and further optimizes the transmission performance of BE messages to improve the overall communication efficiency of the TTE network under given topology and path conditions.

To reduce the overall scheduling complexity, the heterogeneity of network links is identified, and a static scheduling algorithm is independently applied to the critical links. This approach not only shortens the computation time but also optimizes the overall transmission performance of BE messages.

For global network scheduling, due to the NP-hard nature of TTE, traditional methods are prone to local optima or excessive time consumption. Genetic algorithms, with their parallel population search and strong global increment optimization capabilities based on historical population, can quickly approximate the optimal scheduling table. Their flexible encoding makes it easy to incorporate constraints such as time windows and priorities, adapting to multi-objective optimization requirements. Therefore, a genetic algorithm is employed for global network scheduling to leverage its global optimization capability, further enhancing solution quality and reducing computation time.

**Figure 4:**
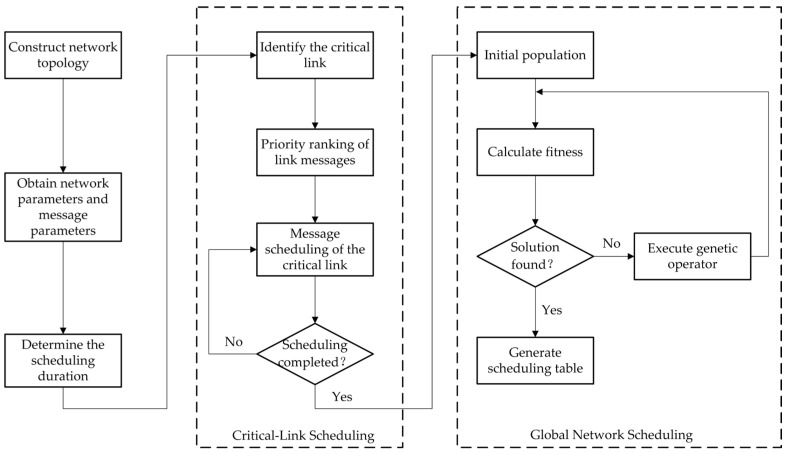
Overall flow of the scheduling algorithm.

Accordingly, the proposed scheduling algorithm adopts a two-stage solution strategy: the first stage focuses on scheduling of the critical link, while the second stage performs global scheduling across the network. The result of the first stage serves as an input constraint for the second stage. The overall flow of scheduling algorithm based on critical-link optimization is illustrated in Figure 4.

First, network and traffic parameters should be obtained based on user transmission requirements, including the network topology, the length, period, duration, and transmission path of all TT messages. The LCM of all TT message periods is then calculated to determine the cluster cycle, which defines the overall scheduling duration.

Then, based on the distribution of messages across network links, the number of messages on each link, the impact of TT messages on the delay and jitter of BE messages, and the structural importance of links are evaluated. Links with high message density, high sensitivity to BE message delay and jitter, or high topological centrality can be selected as critical links. Critical links in different application scenarios can be flexibly selected based on the actual network requirements and the above factors. The detailed method for selecting critical links is described in Section 4.1.2.

Finally, an initial population for TT message scheduling is constructed based on genetic algorithm, in which the scheduling result of the critical link are embedded as fixed genes within each individual. These genes remain unchanged throughout the evolutionary process. Once the initial population is generated, the genetic algorithm iteratively calculates fitness and applies genetic operators until a feasible solution is found, generating the final communication schedule for the TTE.

#### 4.1.2. Critical Link Selection

Link centrality, link traffic load, and link importance are used as three indicators to evaluate link criticality. A quantitative calculation model is constructed to realize the systematic screening of critical links in the network.

Link centrality characterizes the degree of correlation between the location of the link and the location of the core node (the central node or root node in TTE network), and quantifies the topological distance between the link and the core node. The closer the link is to the core node, the higher its centrality in the data transmission path. The centrality index of a link is calculated based on its topological distance to the core node, with a value range of (0, 1]. The calculation formula is as follows:

(14) $$C_{l}=1-\frac{d_{t\_l}}{D_{t}}$$

In the formula, $d_{t\_l}$ represents the topological distance between link l and the core node, and $D_{t}$ represents the maximum topological distance to the core node in the network. When the link is directly connected to the core node, $d_{t\_l}$ can be set to 0, and in which case $C_{l}$ = 1.

Link traffic load represents the actual message transmission pressure borne by a link. The more messages a link carries, the higher its traffic load. The ratio of the number of messages carried by a link to the total number of messages in the entire network is used as the value of link traffic load, with a maximum value of 1. The calculation formula is as follows:

(15) $$L_{l}=\frac{n_{t\_l}}{N_{t}}$$

In the formula, $n_{t\_l}$ represents the actual number of messages carried by link l, and $N_{t}$ represents the total number of messages in the entire network.

Link importance represents the sensitivity of a link to the delay and jitter of BE messages. If the downstream terminal nodes connected by the link (such as industrial sensors and monitoring devices) have stricter requirements on the delay and jitter of BE messages, the sensitivity of the link is higher. During network planning, the link importance of each link in the network is assigned according to application requirements, and a normalized value is used. The calculation formula is as follows:

(16) $$S_{l}=\frac{J_{t}}{j_{t\_l}}$$

In the formula, $j_{t\_l}$ represents the minimum delay or jitter requirement for BE messages by the terminals associated with link l, and $J_{t}$ represents the minimum delay or jitter that the network can provide for BE messages.

To realize the collaborative quantification of the three-dimensional indicators, a weighted sum model is constructed to calculate link criticality. The formula is as follows:

(17) $$LC=\beta_{1}\times C_{l}+\beta_{2}\times L_{l}+\beta_{3}\times S_{l}$$

Among them, $\beta_{1}$, $\beta_{2}$, and $\beta_{3}$ are the weight coefficients of link centrality, link traffic load, and link importance, respectively, satisfying $\beta_{1}+\beta_{2}+\beta_{3}=1$. LC has a value range of (0, 1]; the larger its value, the higher the criticality of the link. Based on the calculation results, the links with high link criticality value can be selected as critical links.

#### 4.1.3. Algorithmic Complexity Analysis

The TTE scheduling problem is essentially a Constraint Satisfaction Problem (CSP) or a Combinatorial Optimization Problem (COP). Its core task is to search for a feasible solution within a large search space. The search space here is defined as all possible combinations of time slot offsets for all frames within the network links. Based on the TTE model introduced earlier, the searchable time domain D for any frame on a link is given by:

(18) $$D=\{0,t_{size},2t_{size},\cdots ,T_{F}-t_{size}\}$$

In this equation, $t_{size}$ denotes a basic time slot unit, $T_{F}$ denotes the cluster cycle. the size of the search space for a single frame on a single link is:

(19) $$d=\frac{T_{F}}{t_{size}}$$

Suppose the network has p links, and each link carries an average of q frames. Then the total number of variables in the network is n=pq, and the original size of the global search space is:

(20) $$S_{ori}=d^{n}$$

This paper applies a static algorithm to quickly fix the scheduling variables of a critical link before solving the global problem. Suppose this link has m frame variables. Then the total number of variables is reduced to n−m. Moreover, due to inter-link scheduling constraints, the fixed schedule of the critical link propagates constraints to other parts of the network, further reducing the search space. Let the average reduction ratio be ρ, where ρ<1. Then, the remaining search space becomes:

(21) $$S_{new}=\rho^{\left(n-m\right)}d^{\left(n-m\right)}={\left(\rho d\right)}^{\left(n-m\right)}$$

The relative reduction ratio of the search space is defined as:

(22) $$R_{S}=\frac{S_{ori}}{S_{new}}=\frac{d^{m}}{\rho^{\left(n-m\right)}}$$

Equation (22) shows that even if ρ is close to 1, as long as m ≥ 1, $R_{S}$ grows rapidly with d and m.

Additionally, after fixing the schedule of one link, the constraint propagation inherent in TTE networks significantly reduces the feasible time windows for upstream and downstream messages. This avoids potential conflicts and eliminates redundant search branches. Although these effects do not directly reduce the number of variables, they greatly improve pruning efficiency and further reduce the actual runtime.

Through the hybrid algorithm optimization proposed in this paper, although the original complexity class of the problem remains unchanged, the global search space is effectively reduced. This leads to a significant decrease in computational complexity and a substantial reduction in the runtime of the algorithm in practical applications.

### 4.2. Critical Link Scheduling

For the critical link, to minimize the impact of consecutive TT messages on BE traffic performance, it is necessary to reserve the maximum possible time gap after each TT message for BE transmission. This ensures that the interference of TT traffic on BE traffic in mixed transmission scenarios is reduced to a minimum—approximately the duration of a single TT frame.

From the perspective of scheduling algorithms, ensuring the determinism of critical link is particularly important, and this paper proposes a time slot balancing scheduling algorithm based on static priorities. First, messages are prioritized according to scheduling difficulty, as messages scheduled later are affected by those already scheduled; thus, messages that are more difficult to schedule should be planned first. After priority sorting is completed, messages are scheduled in descending order of priority based on predefined rules. During scheduling, according to the current time distribution of scheduled messages, the available time gaps are sorted in descending order of inter-frame intervals. Scheduling time is then searched within each gap, starting from the center and sliding outward to both sides in steps of one time slot, until a time point that satisfies the gap constraint and avoids timing conflicts is found, and this time point is then selected as the scheduling point of current message. Compared with SMT and MA, this method is rule-based, and thus the results are deterministic, and the computation time is significantly reduced.

**Figure 5:**
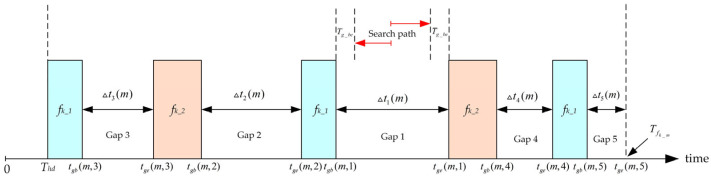
Schematic diagram of the scheduling algorithm.

Assume that link $l_{k}$ is the critical link and $j_{k}$ is its transmitting node. There are $M_{k}$ TT messages to be scheduled on this link, with each message frame denoted as $f_{k\_m}$, and the set of message frames denoted as $F_{k}$. The offset of each message within a cluster cycle is represented by $\Phi_{f_{k\_m}}(i,l_{k},j_{k})$. To ensure the transmission performance of BE messages, a minimum time gap $T_{g\_be}$ is inserted before and after each TT message, and the scheduling results should adhere to this minimum gap constraint. The schematic diagram of the scheduling algorithm is shown in Figure 5.

The figure illustrates the scheduling process of the third message on the critical link. The first two messages result in five available time gaps. When scheduling the third message, the algorithm searches for the best solution in order from Gap 1 to Gap 5, based on descending gap size. The scheduling result of the algorithm can be expressed as follows:

(23) $$\Phi_{f_{k\_0}}(1,l_{k},j_{k})=T_{hd}$$

(24) $$\Phi_{f_{k\_m}}(1,l_{k},j_{k})=\left\{\begin{array}{l} t_{gb}(m,n)+t_{s}(m,n),T_{g\_be}\leq t_{s}(m,n)\leq \Delta t_{n}(m)-T_{g\_be};\\ \forall f_{k\_p}\in F_{k},p=1,2\cdots m-1,i=1,2\cdots N_{f_{k\_p}},j=1,2\cdots N_{f_{k\_m}}\Rightarrow \\ (\Phi_{f_{k\_p}}(i,l_{k},j_{k})\geq \Phi_{f_{k\_m}}(j,l_{k},j_{k}))+\delta_{f_{k\_m}}^{l_{k}}+T_{g\_be}\vee .\\ (\Phi_{f_{k\_m}}(j,l_{k},j_{k})\geq \Phi_{f_{k\_p}}(i,l_{k},j_{k}))+\delta_{f_{k\_p}}^{l_{k}}+T_{g\_be} \end{array}\right.$$

In the equation, $\Phi_{f_{k\_0}}(1,l_{k},j_{k})$ denotes the offset of the first message, $\Phi_{f_{k\_m}}(1,l_{k},j_{k})$ denotes the offset of the m-th message, and $t_{s}(m,n)$ indicates the time offset of the best scheduling point obtained by the algorithms in the n-th gap, relative to the start time $t_{gb}(m,n)$ of that gap. $N_{f_{k\_p}}$ and $N_{f_{k\_m}}$ represent the number of cycles in the cluster cycle of frame $f_{k\_p}$ and $f_{k\_m}$, respectively. $\delta_{f_{k\_p}}^{l_{k}}$ and $\delta_{f_{k\_m}}^{l_{k}}$ represent the transmission duration of frame $f_{k\_p}$ and $f_{k\_m}$ in the link, respectively.

The detailed procedure of the time slot balancing scheduling algorithm based on static priority is as follows:

1. First, the messages on the critical link are sorted by priority. Since messages with shorter periods have higher temporal density and are more difficult to schedule, this paper adopts a period-based priority assignment strategy, in which messages with shorter periods are given higher scheduling priority.
2. The first message on the link is scheduled, and its transmission offset is directly set to the minimum forwarding delay of the switch, denoted as $T_{hd}$, as shown in Equation (23).
3. For the m-th message on the link (m>1), frame gap sorting is performed. Assume that the period of message m is $T_{f_{k\_m}}$. Within the time window $\left(T_{hd},T_{f_{k\_m}}\right)$, suppose there are $N_{g}(m)$ frame gaps formed by the already scheduled messages. These gaps are sorted in descending order according to the size of their intervals. After sorting, the start and end times of the gaps are denoted as $\left(t_{gb}(m,1),t_{ge}(m,1)\right)$, $\left(t_{gb}(m,2),t_{ge}(m,2)\right)$,…, $\left(t_{gb}(m,N_{g}(m)),t_{ge}(m,N_{g}(m))\right)$ and the corresponding gap durations are $\Delta t_{1}(m)$, $\Delta t_{2}(m)$,…, $\Delta t_{N_{g}}(m)$, where $\Delta t_{1}(m)$ > $\Delta t_{2}(m)$ > …… > $\Delta t_{N_{g}}(m).$
4. For the m-th message on the link (m>1), the frame gap for scheduling is determined. After excluding the gaps that have already been searched, the biggest remaining gap is selected as the current gap for scheduling.
5. For the m-th message on the link (m>1), the optimal scheduling time is then searched within the selected frame gap. The search starts from the center of the gap and moves outward to both sides in steps of one time slot. At each step, it checks whether all subsequent occurrences of the message within the cluster cycle satisfy the contention-free constraint described in Section 3.2 and the minimum time gap constraint $T_{g\_be}$. If a best scheduling time is found that meets these constraints, it is selected as the scheduling result, as shown in Equation (24), and the process proceeds to Step 6. If the constraints are not met, the search continues to the next time slot. If no feasible solution is found after searching the entire frame gap, the algorithm returns to Step 4.
6. Determine whether the scheduling result of the m-th message exists in the current solution space. If it does not exist, a new solution is obtained and added to the solution space, and the process proceeds to Step 7. If the result already exists in the solution space, the algorithm returns to Step 5 to continue time slot shifting and search for the next feasible solution.
7. After the m-th message scheduling is completed, check whether $m=M_{k}$. If so, proceed to Step 8; otherwise, increment m by 1 and return to Step 3.
8. Once all messages have been scheduled, a feasible scheduling solution for the critical link, denoted as $\varphi_{n}$, is obtained and added to the set of feasible solutions $\phi_{n}$.
9. Repeat Steps (2) to (8) until the target number of solutions is reached or all feasible solutions have been exhausted, at which point the algorithm terminates.

The above algorithm is for a single critical link. If multiple critical links are identified in the network, the same algorithm process can be applied to each critical link.

### 4.3. Global Network Scheduling

#### 4.3.1. Population Initialization

Before applying the genetic algorithm, an initial population must be generated, and each individual in the population must be initialized. Considering the characteristics of TTE, this paper adopts a decimal encoding scheme. Suppose there are $N_{l}$ links and $N_{f}$ messages to be transmitted in the network. Then, an individual in the population can be represented by an $N_{l}\times N_{f}$ matrix as follows:

(25) $$x_{i}=\left[\begin{array}{c} x_{i\_00}\;\;\;x_{i\_01}\;\;\;\cdots \;\;\;x_{i\_0N_{L}}\\ x_{i\_10}\;\;\;x_{i\_11}\;\;\;\cdots \;\;\;x_{i\_1N_{L}}\\ \;\;\vdots \;\;\vdots \;\;\ddots \;\;\vdots \\ x_{i\_N_{f}0}\;x_{i\_N_{f}1}\;\cdots x_{i\_N_{f}N_{L}} \end{array}\right]$$

In the matrix, $x_{i\_jk}$ represents the time offset of frame j on link k. Since time has been simplified to time slots in the scheduling model, its value is a discrete value starting from 0 with a minimum step size of 1. If frame j does not traverse a particular link, the corresponding encoding value is set to 0, and the link is ignored during computation. The offset values $x_{i\_jk}$ related to the critical link are brought in directly according to the critical link scheduling results in Section 4.2, and their values remain unchanged during subsequent genetic operations.

#### 4.3.2. Fitness Function

The objective function is designed based on the constraints of TTE, including frame period constraint, contention-free constraint, switch traversal constraint and end-to-end delay constraint, and transformed into a fitness function to construct the optimization equation. This optimization equation is subsequently used by genetic algorithm to generate a feasible scheduling table. According to the previously discussed constraints of message scheduling, we can obtain the global TT scheduling table if we determine the offset of each TT message within a single cluster cycle relative to the cycle’s starting time.

1. Frame Period Constraint

For the frame period constraint, the transmission deadline of the frame is used as the objective function. For any frame f, and for any link $l\in {TP}_{f}$ in its path tree, the transmission deadline of the frame can be defined as the following objective function:

(26) $$\varepsilon_{1f}^{l}=\Phi_{f}(1,l,j)+\delta_{f}^{l}$$

If the value of the objective function falls within the interval $\left(0,T_{f}\right]$, the offset of frame f on a given link is considered to meet the frame period constraint, and the corresponding penalty is assigned a value of 0. If the constraint is violated, the penalty is set to $T_{f}-\varepsilon_{1f}^{l}$. Based on this, the fitness function for frame f can be expressed as follows:

(27) $$\varphi_{1f}^{l}=\;\left\{\begin{array}{c} 0,0<\varepsilon_{1f}^{l}\leq T_{f}\\ T_{f}-\varepsilon_{1f}^{l},\varepsilon_{1f}^{l}>T_{f} \end{array}\right.$$

By summing over all TT message frames F and data links L, the overall fitness function $cf_{1}$ under the frame period constraint is obtained, as shown by the following:

(28) $$cf_{1}=\sum_{l}^{L}\sum_{f}^{F}\varphi_{1f}^{l}$$

2. Contention-Free Constraint

For the contention-free constraint, whether a frame meets the contention-free condition is incorporated into the objective function. For any link $(v_{x},v_{y})\in L$, whether the frames $f_{m},f_{n}$ in the link satisfy the contention-free constraint can be defined as the following objective function:

(29) $$\begin{array}{l} \varepsilon_{2\;\;\;m,n}^{\left(v_{x},v_{y}\right)}(a,b)=\left(a\times T_{f_{m}}+\Phi_{f}(1,(v_{x},v_{y}),m)\geq b\times T_{f_{n}}+\Phi_{f}(1,(v_{x},v_{y}),n))+\delta_{f_{n}}^{l}\right)\vee \\ \left(b\times T_{f_{n}}+\Phi_{f}(1,(v_{x},v_{y}),n)\geq a\times T_{f_{m}}+\Phi_{f}(1,(v_{x},v_{y}),m)+\delta_{f_{m}}^{l}\right) \end{array}$$

In the formula, a and b denote the sequence numbers of frames $f_{m}$ and $f_{n}$ on the link, $T_{f_{m}}$ and $T_{f_{n}}$ respectively represent the periods of frames $f_{m}$ and $f_{n}$, $\delta_{f_{m}}^{l}$ and $\delta_{f_{n}}^{l}$ respectively represent the transmission durations of frames $f_{m}$ and $f_{n}$ on the link. If the value of the objective function is 1, it indicates that the transmission interval of frame $f_{m}$ does not overlap with the transmission interval of frame $f_{n}$ on the shared link $\left(v_{x},v_{y}\right)$, meaning no resource conflict occurs and the contention-free constraint is satisfied. In this case, the corresponding penalty is set to 0. Conversely, if the objective function equals 0, it means that $f_{m}$ and $f_{n}$ have overlapping transmission intervals on the link $\left(v_{x},v_{y}\right)$, leading to resource contention, and the associated penalty is denoted as θ. Based on this, the fitness function of frame f can be expressed as follows:

(30) $$\varphi_{2\;\;\;\;m,n}^{\left(v_{x},v_{y}\right)}(a,b)\;=\left\{\begin{array}{c} 0,\;\;\;\;\;\;\;\;\;\;\;\;\;\;\varepsilon_{2\;\;\;\;\;m,n}^{\left(v_{x},v_{y}\right)}(a,b)\neq 0\;\;\;\;\;\\ \theta ,\;\;\;\;\;\;\;\;\;\;\;\;\;\;\varepsilon_{2\;\;\;\;\;m,n}^{\left(v_{x},v_{y}\right)}(a,b)=0\;\;\;\;\; \end{array}\right.$$

Let $A=\frac{T_{F}}{T_{f_{m}}}-1$ and $B=\frac{T_{F}}{T_{f_{n}}}-1$. By summing over all TT message frames F and data links L, the overall fitness function $cf_{2}$ under the non-contention constraint is obtained, as shown by the following:

(31) $$cf_{2}=\sum_{\left(v_{x},v_{y}\right)}^{L}\sum_{f_{m},f_{n}}^{F}\sum_{a=0}^{A}\sum_{b=0}^{B}\varphi_{2\;\;\;\;m,n}^{\left(v_{x},v_{y}\right)}(a,b)\;$$

3. Switch Traversal Constraint

For the switch traversal constraint, the time difference between the transmissions of a TT frame on two adjacent physical paths passing through a switch node is used as the objective function. For any pair of adjacent links $(v_{x},v_{y}),(v_{y},v_{z})\in L$, whether a frame f satisfies the switch traversal constraint can be defined as the following objective function:

(32) $$\varepsilon_{3f}^{\left(\left(v_{x},v_{y}),(v_{y},v_{z}\right)\right)}=\Phi_{f}(i,(v_{y},v_{z}),j)-\Phi_{f}(i,(v_{x},v_{y}),j)$$

Based on the periodic characteristics of TT frames, it is sufficient to satisfy the condition for only the first frame on the link; therefore, the constraint can be simplified as follows:

(33) $$\varepsilon_{3f}^{\left(\left(v_{x},v_{y}),(v_{y},v_{z}\right)\right)}=\Phi_{f}(1,(v_{y},v_{z}),j)-\Phi_{f}(1,(v_{x},v_{y}),j)$$

Based on the relationship between the constraint bounds and the offset difference, if the constraint is satisfied, the penalty is 0; otherwise, the cost value depends on the degree to which the offset deviates from the constraint—the greater the deviation, the higher the cost. Accordingly, the fitness function of frame f quantifies the deviation between the transmission of the TT data frame and the switch traversal constraint, which is defined as follows:

(34) $$\varphi_{3f}^{\left(\left(v_{x},v_{y}),(v_{y},v_{z}\right)\right)}=\left\{\begin{array}{l} 0,\;T_{hd}\;\leq \;\varepsilon_{3f}^{\left(\left(v_{x},v_{y}),(v_{y},v_{z}\right)\right)}\leq \;T_{\mathrm{mm}}\\ T_{hd}\;-\;\varepsilon_{3f}^{\left(\left(v_{x},v_{y}),(v_{y},v_{z}\right)\right)},\;\varepsilon_{3f}^{\left(\left(v_{x},v_{y}),(v_{y},v_{z}\right)\right)}<T_{hd}\\ \varepsilon_{3f}^{\left(\left(v_{x},v_{y}),(v_{y},v_{z}\right)\right)}\;-T_{mm},\;\varepsilon_{3f}^{\left(\left(v_{x},v_{y}),(v_{y},v_{z}\right)\right)}>T_{mm} \end{array}\right.$$

In the formula, $T_{hd}$ represents the minimum time a frame must be stored in the switch before being relayed, and $T_{mm}$ represents the maximum time a frame can be stored in the switch before being forwarded. By summing over all TT data frames F and all switch nodes traversed on their corresponding virtual links $p_{i}$, the overall fitness function $cf_{3}$ under the switch traversal constraint can be obtained, as shown in following:

(35) $$cf_{3}=\sum_{f}^{F}\sum_{\left(v_{x},v_{y}),(v_{y},v_{z}\right)}^{p_{i}}\varphi_{3f}^{\left(\left(v_{x},v_{y}),(v_{y},v_{z}\right)\right)}$$

4. End-to-End Delay Constraint

In the case of multicast transmission, it is assumed that the upper bound of end-to-end delay is the same for all receiving nodes. Based on the constraints described in the previous section, each frame must satisfy that the difference between the offset on the destination link and the offset on the source link falls within the specified delay bound. The objective function is defined as follows:

(36) $$\varepsilon_{4f}=\Phi_{f}(1,(v_{r-1},v_{r}),j)-\Phi_{f}(1,(v_{s},v_{s+1}),j)$$

Here, $\varepsilon_{4f}$ represents the quantized value of the end-to-end delay of a traffic message under the time slot model, which is defined as the difference between the transmission offset on the destination node’s directly connected link and the transmission offset on the source node’s directly connected link. Based on the relationship between the objective function and the constraint deviation, the penalty is zero if the constraint is satisfied; otherwise, the cost depends on how much the offset deviates from the delay bound—the greater the deviation, the higher the cost. Accordingly, the fitness function for frame f is formulated as follows:

(37) $$\varphi_{4f}=\left\{\begin{array}{c} 0,\;\varepsilon_{4f}\leq T_{et}\\ \varepsilon_{4f}-T_{et},\;\varepsilon_{4f}>T_{et} \end{array}\right.$$

In the formula, $T_{et}$ represents the end-to-end delay constraint time. By summing over all TT data frames F, the overall fitness function $cf_{4}$ under the end-to-end transmission delay constraint is obtained, as shown in following:

(38) $$cf_{4\;}=\sum_{f}^{F}\varphi_{4f}$$

5. Optimization Equation Design

By summing the fitness functions corresponding to the aforementioned constraints and assigning the respective weights, the final optimization equation of the genetic algorithm is obtained, as expressed in following:

(39) $$cf=\alpha_{1}\times cf_{1}+\alpha_{2}\times cf_{2}+\alpha_{3}\times cf_{3}+\alpha_{4}\times cf_{4}$$

Here, $\alpha_{1}$, $\alpha_{2}$, $\alpha_{3}$, and $\alpha_{4}$ are constants that adjust the relative weight of the four different constraint-related penalty within the overall fitness function.

#### 4.3.3. Genetic Operators

Genetic operators are the core mechanism of genetic algorithms, and they drive the algorithm to explore the search space and ultimately approximating the global optimal solution by manipulating the genes of individuals in the population. This paper optimizes the design of genetic operators for the TTE scheduling problem, including crossover, mutation, and selection.

This algorithm conducts scheduling based on messages with pre-planned transmission paths. If frames from two different transmission paths are crossovered, the transmission path of the resulting offspring will be changed from the original frames, causing individual failure. Therefore, the crossover is performed only between the same frame across different individuals, and crossover between different frames is avoided to prevent excessive divergence in the search process, which could negatively impact convergence speed. A uniform crossover strategy is adopted, in which 50% of the genes are randomly selected from two parent individuals for exchange. Considering that frame transmission must follow the sequential order of links, the resulting offsets must be re-sorted temporally after crossover to ensure that the link transmission order constraints are met.

Direct manipulation of scheduling time is the core to solving TTE scheduling problems. Therefore, a hybrid mutation mechanism combining time shifting and time swapping is adopted. In the time shifting mechanism, the scheduling time of each frame on each link within an individual is randomly increased or decreased by n time slots. The value of n is selected to ensure that the shifted frame still meets its period constraints. For individuals with larger fitness deviation, a larger n is chosen, while for those with smaller fitness deviation, a smaller n is used. In the time swapping mechanism, the scheduling times of different frames on the same link are randomly exchanged. A new scheduling table is generated, and the mutated frames are re-sorted temporally to conform to the link transmission order.

To improve convergence speed and avoid falling into local optima, a hybrid selection strategy combining parent-offspring competition and random individual competition is adopted. First, in the parent-offspring competition mechanism, both the original individual and its corresponding offspring (after crossover and mutation) are evaluated using the fitness function, and the one with the lower fitness value is selected. This approach preserves high-quality position vectors and accelerates convergence. Second, to enhance population diversity, a random individual competition mechanism is employed. A new individual is randomly generated, and its fitness is compared with that of the original individual. The one with the lower fitness value is retained. This mechanism helps the algorithm escape local optima and expand the search space.

#### 4.3.4. Genetic Operation Process

**Figure 6:**
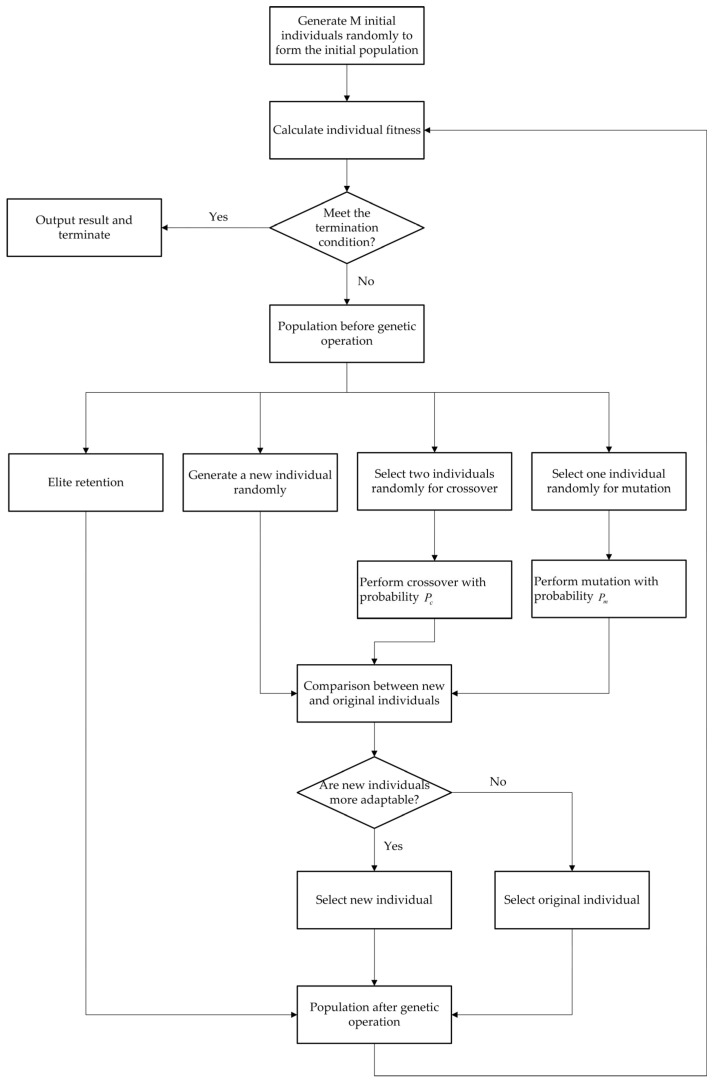
Genetic operation process.

The schedule table of the entire network is solved based on the initial population, fitness function, and genetic operators. The genetic operation process is shown in Figure 6.

M initial individuals are randomly generated to form the initial population. After initialization, the evolution stage begins. The algorithm generates new individuals through three operations: firstly, randomly generating a certain amount of new individuals to increase population diversity; secondly, randomly selecting two individuals and performing crossover operation with probability $P_{c}$; and thirdly, randomly selecting one individual and performing mutation operation with probability $P_{m}$. For each of the above three operations, the fitness of the new and original individuals is compared, and the better individual is selected to enter the next generation population.

During population evolution, the algorithm directly retains a certain proportion of the individuals with higher fitness in the original population through an elite retention strategy.

After completing the genetic operation, calculate the fitness value of each individual in the next generation, and determine whether the termination condition is met. The termination condition is set to obtain a feasible solution or reach a specified solution time. If it is met, output the result and terminate the algorithm; otherwise, use the generated population as the current population and enter the next evolving stage. The entire process iteratively optimizes the population until the termination condition is met, ultimately obtaining a TTE scheduling table that meets the scheduling constraints.

#### 4.3.5. Selection and Analysis of Algorithm Parameters

The main parameters of the genetic algorithm include the population size M, the elite retention ratio r, the fitness function weights $\alpha_{1}$, $\alpha_{2}$, $\alpha_{3}$, $\alpha_{4}$, the initial crossover probability $\omega_{c}$, and the initial mutation probability $\omega_{m}$. For the large-scale TTE network scheduling problem discussed in this paper, the parameter ranges and selection methods are analyzed and explained, along with a sensitivity analysis of these parameters under different network and message scales.

1. Population Size

The population size affects the diversity and convergence speed of the algorithm. The population should be sufficiently large to cover the search space, but an excessively large size will increase computational overhead. For solving the TTE scheduling problem, it is recommended that M be selected within a range of 50 to 600, depending specifically on the problem complexity and available computational resources. In practical applications, fine-tuning based on the algorithm’s runtime performance is also advisable. According to genetic algorithm theory, M should be proportional to the size of the search space. When the network scale and message scale increase, the search space grows exponentially, and M must be increased accordingly.

2. Elite Retention Ratio

The elite retention ratio ensures that superior individuals are preserved for the next generation, influencing both convergence speed and diversity. An excessively high value of r may lead to premature convergence, while an overly low value risks losing high-quality solutions. For solving the TTE scheduling problem, the recommended range for r is between 10% and 20%. According to the theory of elitist strategies, as the network scale and message scale increase, obtaining high-quality solutions becomes more challenging; therefore, r can be appropriately increased to accelerate convergence.

3. Fitness Function Weights

The fitness function weights are used to balance the importance of the frame period constraint, contention-free constraint, switch traversal constraint, and end-to-end delay constraint. In practical applications, frame period constraints and contention-free constraints are the most fundamental constraints for meeting TTE schedulability, and $\alpha_{1}$, $\alpha_{2}$ are usually assigned relatively high weights; the switch traversal constraint is related to the buffering limitations of switches. In practical applications, the buffering capacity of switches is relatively flexible, making it easy to meet the switch traversal constraint conditions for TT frames, so a smaller weight can be assigned to $\alpha_{3}$; the end-to-end delay constraint is a fine adjustment of delay under the above three constraints, and has a smaller impact on overall TTE schedulability, so a smaller weight can also be assigned to $\alpha_{4}$.

For solving the TTE scheduling problem, it is recommended that the values of weights $\alpha_{1}$, $\alpha_{2}$, $\alpha_{3}$, and $\alpha_{4}$ be within the range of [0.4–0.6], [0.2–0.4], [0.05–0.2], and [0.05–0.2], respectively. The values should be positive real numbers and meet the normalization conditions. As the network scale and message scale increase, constraint conflicts intensify, and the weights need dynamic adjustment. $\alpha_{4}$ should increase with scale because delay accumulation becomes more significant in large-scale networks; $\alpha_{2}$ should increase with scale because contention risk becomes more critical; while $\alpha_{1}$ and $\alpha_{3}$ will be relatively stable.

For the optimization of weight values, adjustments can be made based on the initial selected values according to the algorithm’s running results. If it is found that the optimization effect of a certain fitness function is significant while other functions change little, it indicates that the weight of that loss function may be too high, and its weight needs to be appropriately reduced, while the weights of other fitness functions need to be increased, and adjustments are repeated until the optimization effects of all fitness functions tend to be balanced.

4. Crossover and Mutation Probabilities

The crossover and mutation rates control the balance between exploration and exploitation in the algorithm. An excessively high crossover rate may lead to premature convergence, while an overly low rate slows convergence. An excessively high mutation rate can destroy good solutions, while an overly low rate results in insufficient diversity. For solving the TTE scheduling problem, the recommended range for initial crossover probability $\omega_{c}$ is 0.6 to 0.9, and for initial mutation probability $\omega_{m}$ is 0.01 to 0.1. According to classical genetic algorithm theory, $\omega_{c}$ and $\omega_{m}$ should be adjusted according to problem complexity. As the scale increases and the search space expands, enhancing exploration capability becomes necessary; therefore, $\omega_{c}$ can be appropriately increased with scale.

To accelerate the convergence speed of the algorithm, an adaptive parameter tuning method was adopted for the crossover and mutation probabilities, which are tuned according to the following formula:

(40) $$P_{c}=\left\{\begin{array}{l} \omega_{c}\frac{f_{c}-f_{a}}{f_{\max}-f_{a}},\;f_{c}>f_{a}\\ \omega_{c},\;\;\;\;\;f_{c}\leq f_{a} \end{array}\right.$$

(41) $$P_{m}=\left\{\begin{array}{l} \omega_{m}\frac{f-f_{a}}{f_{\max}-f_{a}},\;f>f_{a}\\ \omega_{m},\;\;\;\;\;f\leq f_{a} \end{array}\right.$$

In the formula, $P_{c}$ represents the dynamic crossover probability, $P_{m}$ represents the dynamic mutation probability, $\omega_{c}$ and $\omega_{m}$ represents the initial hyperparameter, $f_{\max}$ represents the maximum fitness value in the population, $f_{a}$ represents the average fitness value in the population, $f_{c}$ represents the fitness value of the parent with higher fitness among the two parents before the crossover operation, and f represents the fitness value of the individual after the mutation operation.

## 5. Simulation and Analysis

### 5.1. Simulation Parameters and Algorithms

In the simulation network, five different types of TT messages are configured. Based on the message periods, the cluster cycle of the network is set to 30,000 time slots. The detailed configuration of the messages is shown in Table 1.

According to the scheduling characteristics of TTE, the greater the number of time slots occupied by TT messages on a link, the higher the scheduling complexity. To quantify the scheduling difficulty of a link, a link scheduling pressure parameter is defined. This parameter is expressed as the ratio between the number of time slots occupied by TT messages on the link within a cluster cycle and the total number of time slots in that period. The link scheduling pressure p of link $l_{k}$ can be calculated as follows:

(42) $$p^{l_{k}}=\frac{\sum_{f}^{F_{k}}\left(\delta_{f}\cdot N_{f}\right)}{T_{F}}$$

In the formula, $T_{F}$ represents the cluster cycle, $N_{f}$ denotes the number of cycles of frame f within the cluster cycle, and $F_{k}$ represents the set of all frames in the critical link. To thoroughly evaluate the scheduling performance of the proposed algorithm under varying scheduling pressures, eight simulation conditions are designed based on different network load levels. These conditions cover a range of scheduling pressure levels on the link, with the number of frames and link scheduling pressure configured for each case shown in Table 2.

The simulation adopts the network topology shown in Figure 2, which includes 35 end systems and 6 switches, totaling 41 network nodes. Frame forwarding behavior occurs only at the switches. The communication links between nodes are represented by straight lines; since the network uses full-duplex bidirectional links, each direct connection represents two separate links. The two numbers on each line indicate the link IDs in both directions. There are 80 links in total across the network. To verify the scalability of the algorithm, the network scale was further increased based on the aforementioned basic network topology, and the configurations of the three resulting simulation network topologies are shown in Table 3.

**Figure 7:**
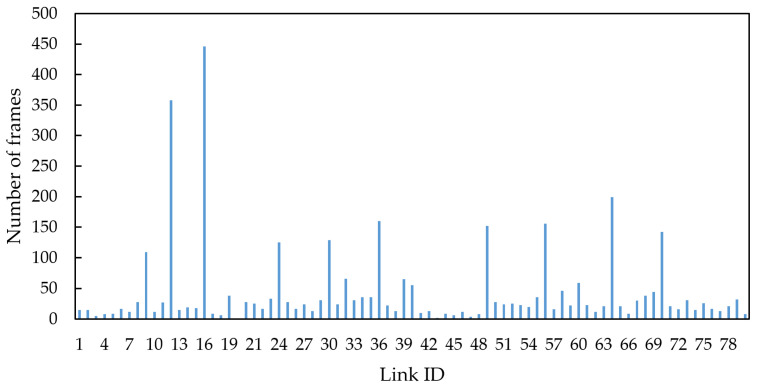
Frame distribution across links under condition 5 and topology 1.

Taking topology 1 as an example, assume that nodes 26–31 have relatively high latency and jitter requirements for BE messages, therefore link 16 associated with them also has high link importance. Meanwhile, due to the direct connection to the central root node, link 16 has the highest link centrality. Finally, we configured link 16 with frames according to Table 2, which has a relatively high link traffic load. The link criticality of each link was calculated, and the link with the highest criticality, link 16, was ultimately selected as the critical link for simulation. The frame distribution across each link under condition 5 and topology 1 is shown in Figure 7.

It can be observed that among the 80 links, there is a significant variation in the frame distribution. Links connected to switch nodes carry more frames compared to those connected to end nodes. Link 16 has the highest frame count and is identified as the critical link in this simulation.

To validate the effectiveness of the proposed algorithm, three scheduling algorithms are selected for comparison: the SMT algorithm (SMTA), the Genetic Algorithm (GA), the Particle Swarm Optimization Algorithm (PSOA) and the proposed Hybrid Scheduling Algorithm (HSA) based on critical-link optimization. Both SMTA and PSOA are based on industrial standard algorithms. GA is the global scheduling algorithm proposed in this paper, with relevant parameters consistent with those of the HSA based on critical link optimization. The weights $\alpha_{1}$, $\alpha_{2}$, $\alpha_{3}$, $\alpha_{4}$ of the fitness function of the genetic algorithm are set to 0.54, 0.32, 0.08, 0.06, respectively, the population size M is set to 350, the elite retention ratio r is set to 20%, the initial crossover probability $\omega_{c}$ is set to 0.8, and the initial mutation probability $\omega_{m}$ is set to 0.08.

The simulation experiments were conducted on a system equipped with an Intel^®^ Core™ i9-12900H CPU and 32 GB of RAM (Intel Corporation, Santa Clara, CA, USA). The scheduling algorithms were implemented using MATLAB R2016a, and comparative simulations were performed on the PyCharm Community Edition 2023.2.3 development platform.

### 5.2. Calculation Time and Success Rate of Solution

Three algorithms were used to solve the TTE scheduling tables under three topologies and eight different conditions, and each algorithm was run 10 times under each condition. If a feasible solution could not be found within 2 h, the run was considered a failure and the simulation was terminated. For the SMT solver, a random seed was set and heuristic search was enabled to ensure the randomness of each solving process. The running results of three algorithms under eight conditions are summarized in Table 4.

In Table 4, each cell contains two values: the average calculation time (in seconds) and the success rate of obtaining a feasible solution. A dash (“–”) indicates that no solution was found within the 2 h time limit.

The comparison shows that in large-scale network TT scheduling, the SMTA has the longest calculation time, which grows rapidly with the increase in message volume, while its success rate continuously declines. Under all three network topologies, there are cases where the SMTA rarely produces feasible results. Since SMTA relies on exhaustive search, while GA and PSOA iteratively optimizes along constraint convergence directions, their solving times are relatively shorter than that of SMTA, and their success rates are higher. However, as message scale increases, there are cases where no solution can be found for GA under all three topologies, and for PSOA under topologies 2 and 3. Compared to SMTA, GA and PSOA, the proposed HSA consistently achieves the shortest solving times across all conditions and succeeds in all topologies. This improvement stems from combining the critical link algorithm with a global genetic algorithm, which enhances the efficiency of scheduling on key links and overall solution quality, making it better suited for large-scale message transmission in complex topologies.

### 5.3. Degree of Balance in Message Scheduling

Since the scheduling balance of TT messages directly affects the performance of BE messages, the scheduling balance achieved by the three algorithms is compared using the formula defined as follows:

(43) $$E=1-\frac{\sum_{i=1}^{n}\sum_{j=1}^{n}\left|g_{i}-g_{j}\right|}{2n^{2}\bar{g}}$$

**Figure 8:**
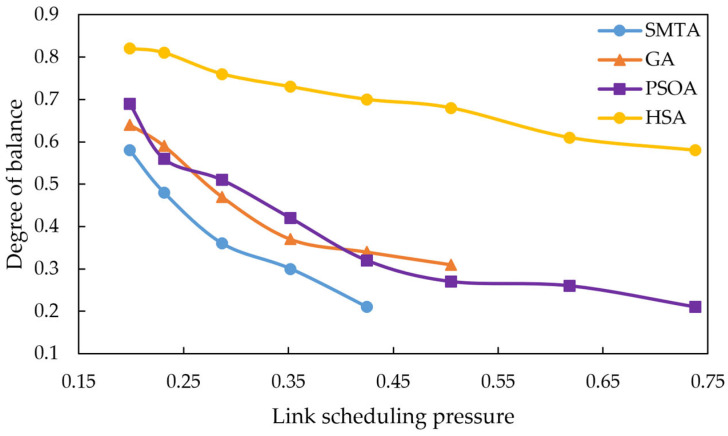
Average scheduling balance.

Here, $g_{i}$ denotes the time interval between any two TT messages, n represents the total number of message intervals, and $\bar{g}$ denotes the average message interval. The calculated degree of balance ranges from 0 to 1, where a larger value indicates higher balance. The average scheduling balance of the three algorithms under eight conditions is shown in Figure 8.

The comparison of scheduling balance above shows that HSA consistently achieves higher balance than the SMTA, GA and PSOA across all conditions. As the message scale increases, the SMTA, GA and PSOA exhibit a significant decline in balance, whereas the HSA maintains a relatively stable balance level. This stability results from the HSA’s use of a slot-balanced scheduling algorithm for the critical link during the initial static scheduling phase, which ensures an even distribution of TT message intervals on the critical link. Additionally, due to the periodic nature of TT messages, this balanced distribution on the critical link propagates throughout the entire network, thereby optimizing overall network balance.

**Figure 9:**
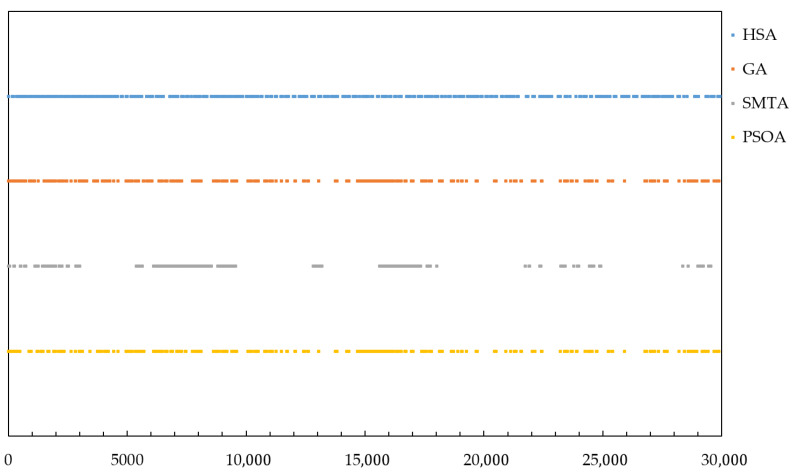
Gantt charts of TT frames generated by the three algorithms under condition 5.

Taking condition 5 under topology 1 as an example, the Gantt charts of TT frames of link 16 generated by the three algorithms are shown in Figure 9.

Figure 9 shows that the message scheduling obtained by the SMTA exhibits high randomness, resulting in the most uneven slot distribution and clearly causing greater delay and jitter for BE messages. The GA and PSOA produces a relatively more uniform message distribution compared to SMTA. The proposed HSA achieves the most optimal slot distribution, with well-spaced intervals between TT messages, minimizing the impact on BE message transmission performance.

### 5.4. BE Message Transmission Performance

**Figure 10:**
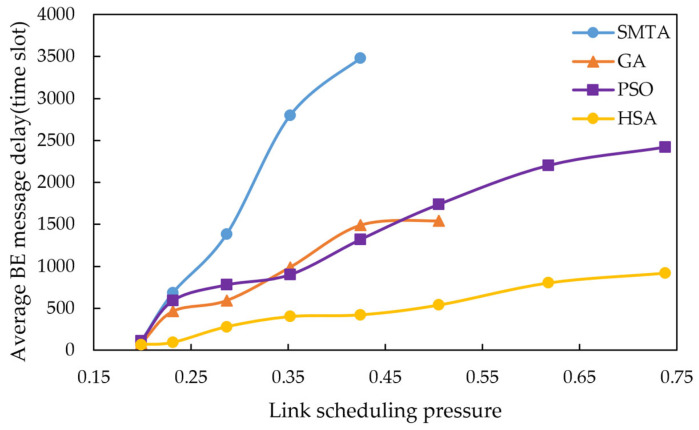
Average BE message delay under topology 1.

**Figure 11:**
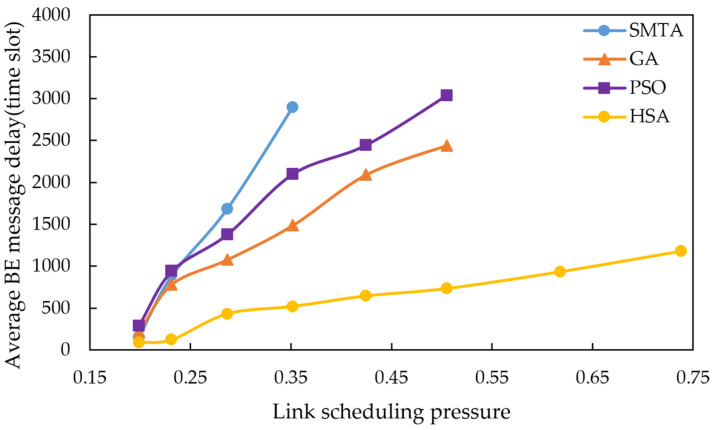
Average BE message delay under topology 2.

**Figure 12:**
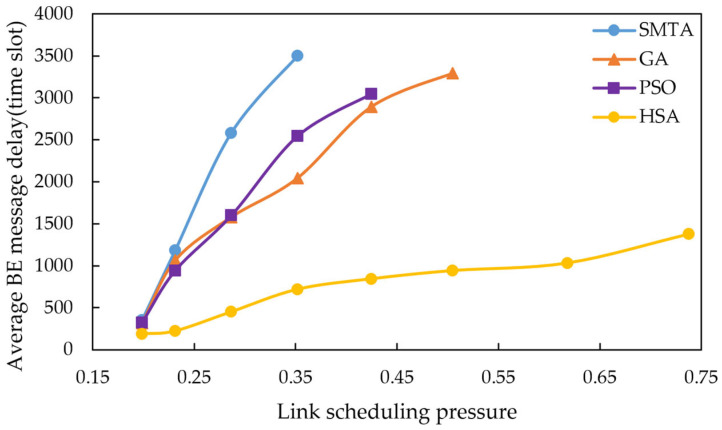
Average BE message delay under topology 3.

**Figure 13:**
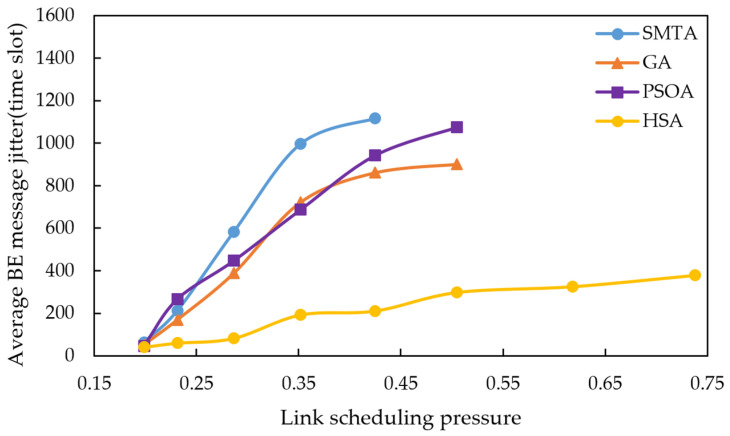
Average BE message jitter under topology 1.

**Figure 14:**
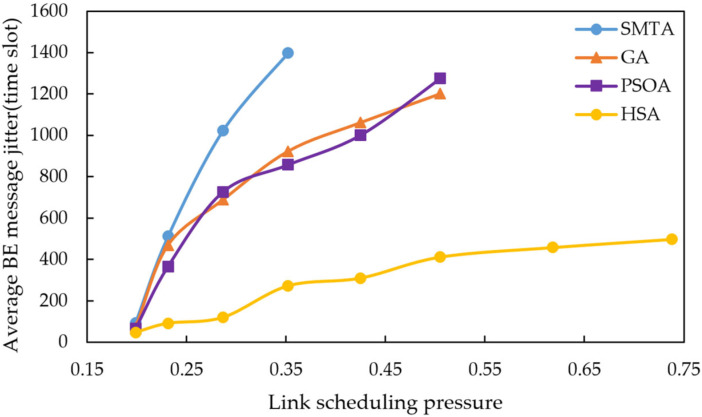
Average BE message jitter under topology 2.

**Figure 15:**
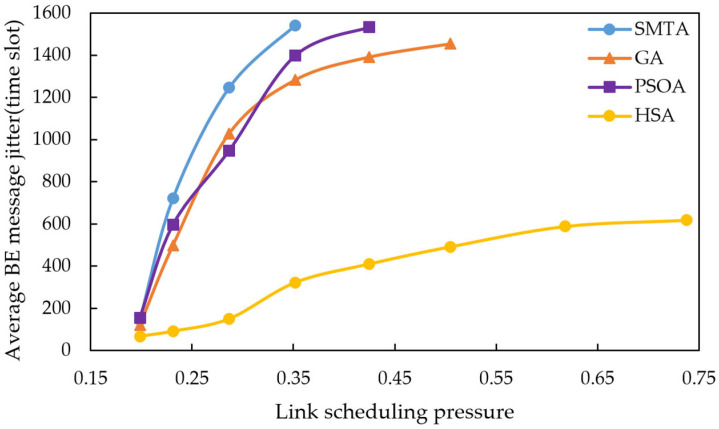
Average BE message jitter under topology 3.

Using the scheduling results described above, a simulation of network message transmission performance was conducted. Four hundred BE messages were randomly generated from network nodes, and their transmission delays were recorded at the receivers. To simulate random network traffic, the arrival process of BE messages is set to a Poisson process and the packet-size distribution is set to log-normal distribution. The average BE message delay under the eight conditions for different algorithms are shown in Figure 10, Figure 11 and Figure 12, with the corresponding average jitter presented in Figure 13, Figure 14 and Figure 15.

The figures indicate that as the message scale increases, the BE message delay and jitter resulting from the scheduling table of SMTA, GA and PSOA grow significantly, whereas those from the HSA remain substantially lower. For instance, under topology 1 and condition 5, the average BE message delay with the HSA is reduced by 87.9% compared to SMTA, by 71.8% compared to GA, and by 68.1% compared to PSOA. Similarly, the average BE message jitter with the HSA decreases by 81.1% relative to SMTA, by 75.6% relative to GA, and by 77.7% relative to PSOA.

**Figure 16:**
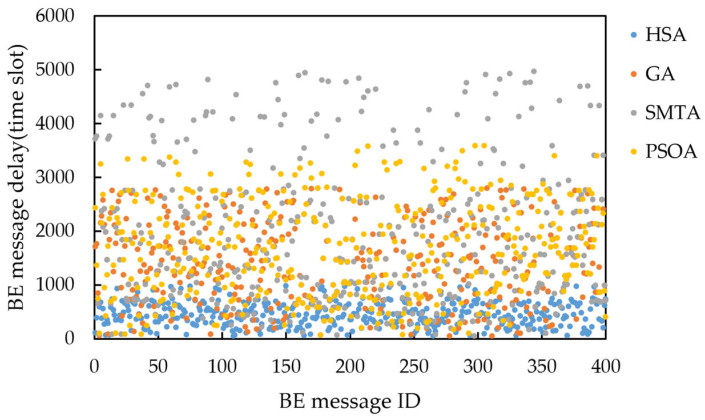
Delay distribution of four hundred BE messages under condition 5 and topology 1.

**Figure 17:**
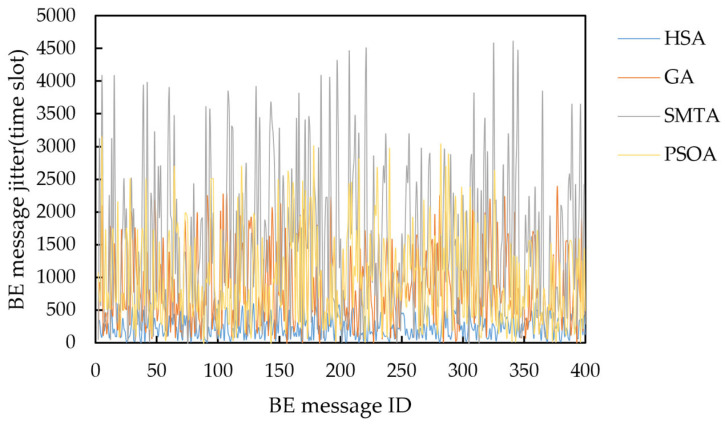
Jitter distribution of four hundred BE messages under condition 5 and topology 1.

Taking topology 1 and condition 5 as an example, the delay distribution of four hundred BE messages under the scheduling results of the three algorithms is shown in Figure 16, and the jitter distribution is shown in Figure 17.

The figure shows that the scheduling results from the SMTA, GA and PSOA exhibit significant fluctuations in BE message transmission delay due to the “back-to-back” arrangement of numerous TT messages. Specifically, the SMTA’s maximum delay and jitter are 4970 and 4689 time slots, respectively; the GA’s maximum delay and jitter are 2800 and 2391 time slots respectively; and the PSOA’s maximum delay and jitter are 3585 and 3151 time slots respectively. In contrast, the HSA employs static scheduling based on slot balancing on the critical link, producing deterministic schedules that facilitate rapid BE message transmission through gaps between TT messages. As a result, HSA achieves the lowest message delays, with a maximum delay of 980 time slots and a maximum jitter of 598 time slots. This demonstrates that the HSA significantly reduces BE message transmission delay and jitter.

## 6. Conclusions

This paper addresses the scheduling demands of large-scale TTE by constructing a representative large-scale TTE network topology and establishing a TTE message scheduling model. The scheduling constraints of TTE messages are analyzed, and a critical-link optimized scheduling algorithm for large-scale TTE is proposed. The algorithm adopts a two-step approach: first, it applies a static priority–based slot-balanced scheduling algorithm on critical links; then, it incorporates the critical link results as fixed genes in the initial population of a genetic algorithm to perform global network scheduling. This method optimizes both the solving process and the results. As the network message volume increases, the algorithm improves the success rate, reduces solving time, and effectively minimizes the impact of TT messages on BE message transmission performance, making it well-suited for large-scale TTE scheduling scenarios. The following conclusions can be drawn:

Compared to traditional algorithms that apply the same solving method to all links, this work identifies link heterogeneity by locating critical links within the network topology and applies a distinct static solving algorithm specifically for these critical links, thereby improving solving efficiency. Additionally, the use of slot-balanced scheduling on critical links effectively reduces the impact of the scheduling results on BE message performance.

Compared to the conventional SMT algorithm used in engineering applications, this paper employs a genetic algorithm to solve the global network scheduling table. It abstracts network constraints into a mathematical model and constructs a scheduling framework with effective fitness functions and genetic operators. Leveraging the genetic algorithm’s strong global search capability and parallel computing advantages, the approach accelerates convergence and enhances scheduling performance.

Simulation results demonstrate that, in large-scale TTE environments, the proposed algorithm achieves higher solving success rates and shorter solving times compared to traditional methods. Additionally, it effectively reduces BE message transmission delay and jitter, making it more suitable for dense message scheduling in large-scale, complex network topologies.

Despite the insights provided, several limitations merit mention. Firstly, the algorithm we proposed is a static solution and offline scheduling algorithm. In industrial networks, there may be application scenarios where network topology or traffic changes dynamically. Future work will focus on the incremental or online scheduling methods of TTE. Secondly, the algorithm proposed in this paper is based on the premise that the transmission path of messages has been pre-determined. Factors such as path selection, path backup, and path distance constraints in path planning also affect the scheduling results of messages [30]. In subsequent work, we will continue to study the joint optimization of message path planning and message scheduling.

## Figures and Tables

**Table 1:** Detailed configuration of the messages.

Message Name	Message Period (Time Slot)	Message Frame Duration (Time Slot)	Number of Frames per Message Within LCM	Total Message Frame Duration in LCM (Time Slot)
Message 1	2000	10	15	150
Message 2	3000	30	10	300
Message 3	5000	40	6	240
Message 4	10,000	100	3	300
Message 5	15,000	70	2	140

**Table 2:** Configuration of eight simulation conditions.

Condition	Number of Frames Within the Critical Link	Number of Frames Within the Total Network	Total Message Frame Duration within the Critical Link	Link Scheduling Pressure of the Critical Link
Condition 1	169	1504	5960	0.199
Condition 2	217	1710	6940	0.231
Condition 3	288	1916	8600	0.287
Condition 4	357	2122	10,560	0.352
Condition 5	446	2328	12,740	0.425
Condition 6	533	2534	15,150	0.505
Condition 7	614	2740	18,540	0.618
Condition 8	698	2946	22,140	0.738

**Table 3:** Configuration of three simulation network topologies.

Network Topology	Number of Endpoints	Number of Switches	Number of Links	Number of Frames Within the Total Network							
				Cond. 1	Cond. 2	Cond. 3	Cond. 4	Cond. 5	Cond. 6	Cond. 7	Cond. 8
Topology 1	35	6	80	1504	1710	1916	2122	2328	2534	2740	3146
Topology 2	150	16	164	3348	4154	4996	5774	6562	7368	8204	10,126
Topology 3	320	25	344	9426	12,926	15,384	16,260	19,062	23,876	28,094	31,248

**Table 4:** Running results of three algorithms under three topologies and eight conditions.

Condition	Running Results											
	SMTA			GA			PSOA			HSA		
	Topo. 1	Topo. 2	Topo. 3	Topo. 1	Topo. 2	Topo. 3	Topo. 1	Topo. 2	Topo. 3	Topo. 1	Topo. 2	Topo. 3
Condition 1	33	376	569	10	44	174	6	912	1467	4	12	46
	100%	100%	100%	100%	100%	100%	100%	100%	100%	100%	100%	100%
Condition 2	94	395	691	27	327	951	10	3724	5981	11	267	298
	100%	100%	100%	100%	100%	100%	100%	80%	70%	100%	100%	100%
Condition 3	869	4526	5412	402	2645	3197	198	4648	6804	44	325	666
	80%	70%	70%	100%	100%	100%	100%	50%	50%	100%	100%	100%
Condition 4	6681	7197	6986	1055	5719	6834	1513	6674	6955	70	514	1051
	40%	20%	10%	80%	70%	60%	100%	30%	20%	100%	100%	100%
Condition 5	7035	–	–	3289	6725	7023	3717	6935	7035	90	657	1471
	10%			70%	50%	20%	70%	10%	10%	100%	100%	100%
Condition 6	–	–	–	5190	6935	7097	4647	7103	–	113	874	2067
				50%	20%	10%	40%	10%		100%	100%	100%
Condition 7	–	–	–	–	–	–	4974	–	–	436	954	2584
							30%			100%	100%	100%
Condition 8	–	–	–	–	–	–	6269	–	–	1259	1964	3165
							30%			100%	100%	100%

## Data Availability

The original contributions presented in this study are included in the article. Further inquiries can be directed to the corresponding author.

## Abbreviations

The following abbreviations are used in this manuscript:

BAG	Bandwidth Allocation Gap
BE	Best-Effort
CSP	Constraint Satisfaction Problem
DPP	Dynamic Programming Priority
ET	Event-Triggered
FIFO	First In, First Out
FQPSO	Fuzzy-controlled Quantum-behaved Particle Swarm Optimization
GA	Genetic Algorithm
HSA	Hybrid Scheduling Algorithm (based on critical-link optimization)
IIoT	Industrial Internet of Things
LCM	least common multiple
MA	Metaheuristic Algorithms
MWRR	Modified Weighted Round-Robin
NPC	NP-Complete Problem
PSOA	Particle Swarm Optimization Algorithm
RC	Rate-Constrained
SA	Simulated Annealing
SMTA	Satisfiability Modulo Theory Algorithm
SMT	Satisfiability Modulo Theory
TTE	Time-Triggered Ethernet
TT	Time-Triggered

## References

[1] Babayigit B., Abubaker M. (2024). Industrial Internet of Things: A Review of Improvements over Traditional SCADA Systems for Industrial Automation. IEEE Syst. J..

[2] Xie X., Wang H., Liu X. (2024). Scheduling for Minimizing the Age of Information in Multisensor Multiserver Industrial Internet of Things Systems. IEEE Trans. Ind. Inform..

[3] Steiner W., Bauer G., Hall B., Paulitsch M., Varadarajan S. TTEthernet dataflow concept. Proceedings of the Eighth IEEE International Symposium on Network Computing and Applications.

[4] Song Y., Zhou C., Luo Y., Huang J. Research on Synchronization Mechanism of Time Triggered Ethernet. Proceedings of the 2024 International Conference on Guidance, Navigation and Control (ICGNC 2024).

[5] Khanmohamadi M., Guerrieri M. (2025). Smart Intersections and Connected Autonomous Vehicles for Sustainable Smart Cities: A Brief Review. Sustainability.

[6] Chen C., Sun Y., Sun Z., Wang D., He X., Cheng B., Liu Y., Yin Y. (2024). Research on the Deterministic Ethernet Application for Manned Lunar Exploration. J. Astronaut..

[7] TARIQ N., PETRUNIN I., AL-RUBAYE S. Analysis of Synchronization in Distributed Avionics Systems Based on Time-Triggered Ethernet. Proceedings of the 2021 IEEE/AIAA 40TH Digital Avionics Systems Conference (DASC).

[8] ZHANG X., ZHAO X. (2020). Architecture design of distributed redundant flight control computer based on time-triggered buses for UAVs. IEEE Sens. J..

[9] Lu J., Xiong H., He F., Zheng Z., Li H. (2020). A Mixed-Critical Consistent Update Algorithm in Software Defined Time-Triggered Ethernet Using Time Window. IEEE Access.

[10] Calabrese M., Curbo J., Falco G. A Software Defined Networking Architecture for Time Triggered Ethernet in Space Systems. Proceedings of the 2024 IEEE International Conference on Wireless for Space and Extreme Environments (WiSEE).

[11] Ye F., Chen Y., Wang T., Ji Y., Luo M., Jiang X. (2022). A load-balanced TTE scheduling method for large-scale message transmission. J. Sichuan Univ..

[12] Chen C., Zhao A., Zhang Z., Zhang T., Fan C. (2025). Research on Multi-Agent Collaborative Scheduling Planning Method for Time-Triggered Networks. Electronics.

[13] Zhang X., Fan Y., Zhou S. (2025). Research progress on Time-Triggered Ethernet traffic scheduling. Telecommun. Eng..

[14] Steiner W. An Evaluation of SMT-Based Schedule Synthesis for Time-Triggered Multi-hop Networks. Proceedings of the Real-Time Systems Symposium.

[15] Song Z., Li Q., Wang J., Xiong H. (2018). Time-triggered scheduling table generation method based on schedulability ranking. J. Beijing Univ. Aeronaut. Astronaut..

[16] Wei A., Zhang G., Zhang T. Research on time triggered ethernet scheduling planning method. Proceedings of the 2020 4th International Conference on Machine Vision and Information Technology (CMVIT 2020).

[17] Jian J., Wang L., Chen H., Nie X. (2020). Scheduling optimization of time-triggered cyber-physical systems based on fuzzy-controlled QPSO and SMT solver. Energies.

[18] Yuan H., Wang Y. A Hybrid Schedule Technology Based on Genetic Algorithm and Simulated Annealing for Time-Triggered Ethernet. Proceedings of the 2022 IEEE 2nd International Conference on Information Communication and Software Engineering (ICICSE).

[19] Zhang Y., He F., Lu G., Xiong H. (2017). Scheduling Rate-Constrained Flows with Dynamic Programming Priority in Time-Triggered Ethernet. Chin. J. Electron..

[20] Zhang Y., He F., Lu G., Xiong H. (2017). A modified weighted round robin scheduling algorithm in TTE. J. Beijing Univ..

[21] Finzi A., Craciunas S. Integration of SMT-based scheduling with RC network calculus analysis in TTEthernet networks. Proceedings of the 2019 24th IEEE International Conference on Emerging Technologies and Factory Automation (ETFA).

[22] Yuan H., Wang Y. (2023). A Time-Triggered Ethernet Optimal Scheduling Technology Based on Rapid Increment. Electron. Opt. Control..

[23] Dvořák J., Heller M., Hanzálek Z. Makespan minimization of Time-Triggered traffic on a TTEthernet network. Proceedings of the 2017 IEEE 13th International Workshop on Factory Communication Systems (WFCS).

[24] Lu Y., Xiong X., Wang M., Qin J., Pan W. (2023). A Bandwidth Allocation Method of AVB Traffic Based on Link Load Balancing in TSN. J. South China Univ. Technol..

[25] Moutsinas G., Guo W. (2020). Probabilistic Stability of Traffic Load Balancing on Wireless Complex Networks. IEEE Syst. J..

[26] Falk J., Dürr F., Rothermel K. Time-triggered traffic planning for data networks with conflict graphs. Proceedings of the 2020 IEEE Real-Time and Embedded Technology and Applications Symposium.

[27] Liu M., Yin H., Li H., Ji X. An efficient scheduling algorithm for adjustable time slots in time-triggered ethernet. Proceedings of the 2019 IEEE 11th International Conference on Communication Software and Networks (ICCSN).

[28] Eramo V., Fiori T., Lavacca F.G., Valente F., Baiocchi A., Ciabuschi S., Albano M., Cavallini E. (2023). A max plus algebra based scheduling algorithm for supporting time triggered services in ethernet networks. Comput. Commun..

[29] Wang B., Han S., Zhang J., Wu J., Meng W. Research of Telemetry Communication Technology Based on TTE Time Triggered Ethernet. Proceedings of the 2023 IEEE International Symposium on Broadband Multimedia Systems and Broadcasting (BMSB).

[30] Arbelaez A., Mehta D., O’Sullivan B., Quesada L. (2018). A constraint-based parallel local search for the edge-disjoint rooted distance-constrained minimum spanning tree problem. J. Heuristics.

